# Miscellaneous Notices

**Published:** 1852-10-01

**Authors:** 


### Miscellaneous Notices.

---

*The Stomach and its Difficulties.* By Sir JAMES EYRE, M.D. Churchill. 1852.

THERE are few things connected with literature so difficult to accomplish as that of writing a pleasant and readable work on a medical subject. The market is over-stocked with dull, heavy, prosy works, which few have the patience to read. It is gratifying to meet with a member of the profession capable of placing before us a volume connected with a most important class of affections, written like the one now upon our table. Sir James Eyre takes no elevated flights; he leaves the more abstruse points in relation to this subject to such men as Dr. W. Philip, and others, and confines his attention to the consideration of those matters which admit legitimately of a somewhat extra-professional discussion. In this respect he has followed in the wake of the late Dr. James Johnson, whose valuable works on indigestion and health are as much addressed to the public as to the profession. Sir James Eyre has the ability to communicate valuable knowledge in an agreeable manner. This little volume is as amusing as a novel, and yet it is replete with facts and principles of the utmost importance to the preservation of the human health. Thousands who could not be persuaded to peruse the recognised medical text-books on the subjects of diet, and indigestion, will eagerly devour the contents of Sir James Eyre's little volume; and if they are wise enough to follow the sensible advice he offers for the regulation of the digestive apparatus, how much misery they will be exempted from. It would be unfair, and manifestly unjust, to subject a work like the present to serious criticism. Sir James Eyre does not put his little volume forward with any ostentatious pretensions. It conveys what he intended it to convey, sensible and practical instructions for the regulation of the functions of the stomach, adapted for general perusal. The little volume is full of pleasantries, all embodying, however, an important principle of treatment.

*Rheumatism, Gout, and Neuralgia as affecting the Head and Ear, &c. &c.* By WILLIAM HARVEY, Surgeon to the Royal Dispensary for Diseases of the Ear.

MR. HARVEY has devoted his attention for many years to aural medicine and surgery; and the well-earned reputation he has acquired, entitles anything that proceeds from his pen to the careful consideration of the profession. The volume under review is an excellent work on several important diseases, written in a clear style, and full of sound practical observations.

*On the Diseases of the Bladder.* By W. COULSON, Esq., Surgeon to St. Mary's Hospital. 4th Edition. Churchill. 1852.

MR. COULSON's work requires no commendation from us to recommend it to the notice of the profession. It is the most comprehensive, practical, and valuable work on the subject of which it treats. The edition before us embodies all the most recent discoveries of any importance connected with the diseases of the bladder; and these, combined with Mr. Coulson's own original remarks, add great value to his work.

*On the Nature and Treatment of the Diseases of the Heart.* By JAMES WARDROP, M.D.

THE principal portion of this work has been published in the columns of the *Medical Times and Gazette.* We have read Dr. Wardrop's volume with much interest. It is a valuable contribution to medical literature. There are many passages in the work of a psychological character, which we had marked for quotation; but we regret that want of space compels us to set them for the present aside. This work must find its way into every medical man's library, and be referred to by all anxious to make themselves acquainted with the treatment of diseases of the heart.

*Poems, Essays, and Opinions.* By ALFRED BATES RICHARDS, Esq., Barrister-at-Law. 4 vols. 1852.

MR. RICHARDS is a vigorous writer. He handles the "grey goose-quill" with considerable ability. His able tragedy, *Crœsus, King of Lydia*, had fully prepared us for the advent of a man of original capacity, and one who dared to think for himself. All the essays are on topics of great, of popular, and scientific interest. It is with much pleasure that we recommend these volumes to the notice of our readers.

*A Practical Treatise on Diseases of the Skin.* By J. MOORE NELIGAN, M.D., M.R.I.A., &c. 1 vol. 8vo. Dublin: Fannin and Co.

WE have been much pleased with this volume. Dr. Neligan writes with all the ease and confidence of a man taught in a practical school. His work is a valuable addition to the literature of skin diseases. We predict for it an extensive sale and great popularity. Dr. Neligan's work may be considered one of the best practical works extant on the diseases of the skin.

